# Sugarcane/Soybean Intercropping with Reduced Nitrogen Application Synergistically Increases Plant Carbon Fixation and Soil Organic Carbon Sequestration

**DOI:** 10.3390/plants13162337

**Published:** 2024-08-22

**Authors:** Tantan Zhang, Yali Liu, Lin Li

**Affiliations:** 1 College of Chemistry and Bioengineering, Hunan University of Science and Engineering, Yongzhou 425199, China; 2 Hunan Engineering Technology Research Center for Comprehensive Development and Utilization of Biomass Resources, Yongzhou 425199, China; 3 College of Natural Resources and Environmental, South China Agricultural University, Guangzhou 510642, China; liuyali5699@126.com; 4 College of Breeding and Multiplication (Sanya Institute of Breeding and Multiplication), Hainan University, Sanya 572000, China

**Keywords:** sugarcane/soybean intercropping, microbial biomass carbon, dissolved organic carbon, plant C fixation

## Abstract

Sugarcane/soybean intercropping and reduced nitrogen (N) application as an important sustainable agricultural pattern can increase crop primary productivity and improve soil ecological functions, thereby affecting soil organic carbon (SOC) input and turnover. To explore the potential mechanism of sugarcane/soybean intercropping affecting SOC sequestration, a two-factor long-term field experiment was carried out, which included planting pattern (sugarcane monocropping (MS), sugarcane/soybean 1:1 intercropping (SB1), and sugarcane/soybean 1:2 intercropping (SB2)) and nitrogen addition levels (reduced N application (N1: 300 kg·hm^−2^) and conventional N application (N2: 525 kg·hm^−2^)). The results showed that the shoot and root C fixation in the sugarcane/soybean intercropping system were significantly higher than those in the sugarcane monocropping system during the whole growth period of sugarcane, and the N application level had no significant effect on the C fixation of plants in the intercropping system. Sugarcane/soybean intercropping also increased the contents of total organic C (TOC), labile organic C fraction [microbial biomass C (MBC) and dissolved organic C (DOC)] in the soil during the growth period of sugarcane, and this effect was more obvious at the N1 level. We further analyzed the relationship between plant C sequestration and SOC fraction content using regression equations and found that both plant shoot and root C sequestration were significantly correlated with TOC, MBC, and DOC content. This suggests that sugarcane/soybean intercropping increases the amount of C input to the soil by improving crop shoot and root C sequestration, which then promotes the content of each SOC fraction. The results of this study indicate that sugarcane/soybean intercropping and reduced N application patterns can synergistically improve plant and soil C fixation, which is of great significance for improving crop yields, increasing soil fertility, and reducing greenhouse gas emissions from agricultural fields.

## 1. Introduction

With the development of the industrial revolution, human activities continue to emit a large number of greenhouse gases, leading to a concentration of CO_2_ in the atmosphere reaching 420 ppm by 2023 [1]. The continuous increase of CO_2_ concentration will lead to the imbalance of the Earth’s energy, which in turn will trigger global climate change [2]. Therefore, the issue of C sequestration and emission reduction has become the focus of attention of governments. According to a report by the Food and Agriculture Organization of the United Nations, about 21% of global greenhouse gases come from agricultural emissions [3]. As the largest C pool in the farmland ecosystem, the soil C pool has the potential to alleviate climate change, and it can also affect soil nutrient cycling, soil ecological balance, and crop growth. Increasing soil organic C sequestration is not only conducive to global greenhouse gas mitigation but also has important significance for improving crop yield and promoting sustainable agricultural development [4,5]. However, at a certain time and space scale, whether the farmland ecosystem can become a greenhouse gas sink or source depends largely on the extent of human disturbance and management of the ecosystem. Therefore, it is of great significance to study the C fixation law of farmland crops and soil for scientific evaluation and seeking farmland management measures to improve soil C fixation.

N fertilizer application is the main management practice to increase crop yield in modern agriculture. Increasing N fertilizer can not only promote plant growth but also strongly change the fate of photosynthetic C in the plant–soil system [6,7]. N application promotes the increase of chlorophyll content in leaves, thereby increasing the photosynthetic rate of plants, and further affects the input of photosynthetic C to plants and its transport to roots and soil [8,9]. The availability of N in soil also affects the mineralization of photosynthetic C in rhizosphere sediments by affecting the diversity and abundance of microbial communities, thus further affecting the C sequestration in soil [10,11]. Existing studies have shown that plants grown in N-deficient environments increase the proportion of C allocated to roots to promote N uptake, and the increase in N availability results in more C allocated to shoot tissues [12].

Plant diversity is also an important factor influencing photosynthetic C allocation. Different plant species coexist naturally, and interspecific competition is widespread. Plants usually adopt the strategy of investing more C in functional organs such as roots in order to compete for limited resources [13,14]. Therefore, there is a feedback interaction between plant diversity and underground C allocation [15]. Forrester et al. [16] used the mass balance method to find that the proportion of C in the root system increased under the mixed cultivation mode of *Eucalyptus globulus* and sponge tree (*Vachellia farnesiana* L.) compared with the single cultivation mode. Similar results were found in grassland ecosystems; that is, the C input into the soil increased when the grassland plant diversity increased [17]. These results indicated that plant diversity could affect the allocation of belowground C in plants. In the farmland intercropping system, there is also interspecific complementation and competition among different crops. Plant aboveground complementarity promotes light interception and utilization efficiency through differences in crop height and light demand [18,19], while belowground complementarity increases water and nutrient acquisition through niche differentiation and resource allocation. In addition to complementarities between different crops, competition for light, water, and nutrients is inevitable due to limited resources [20,21]. The competition between aboveground and belowground resources also affects the distribution of photosynthetic C in the plant–soil system.

Sugarcane (*Saccharum officinarum* L.)/soybean (*Glycine max* L.) intercropping and reduced N application has been utilized for thousands of years in China as a cropping pattern that can improve land utilization, reduce resource wastage, and increase crop yields, and in recent years it has been widely promoted in South China [22]. Many studies have shown that gramineous/leguminous intercropping patterns can promote plant biomass accumulation, such as barley (*Hordeum vulgare* L.)/soybean intercropping [23] and maize (*Zea mays* L.)/alfalfa (*Medicago sativa* L.) intercropping [24]. The ultimate cause of increased plant biomass is the result of photosynthetic C fixation. However, there are few studies on plant C fixation and soil C sequestration in farmland intercropping ecosystems. The objectives of this study were to explore the internal relationship between the change of plant C fixation and SOC turnover by tracking the changes in C sequestration of plant shoots and roots and the SOC content during the growth of sugarcane and soybean. This study provides a basis for further understanding the effect of sugarcane/soybean intercropping on soil C sequestration and a reasonable evaluation of the C budget balance and C sequestration potential of the intercropping system.

## 2. Results

### 2.1. Dynamic Changes of C Sequestration in the Shoots and Roots of Sugarcane

**Figure 1:**
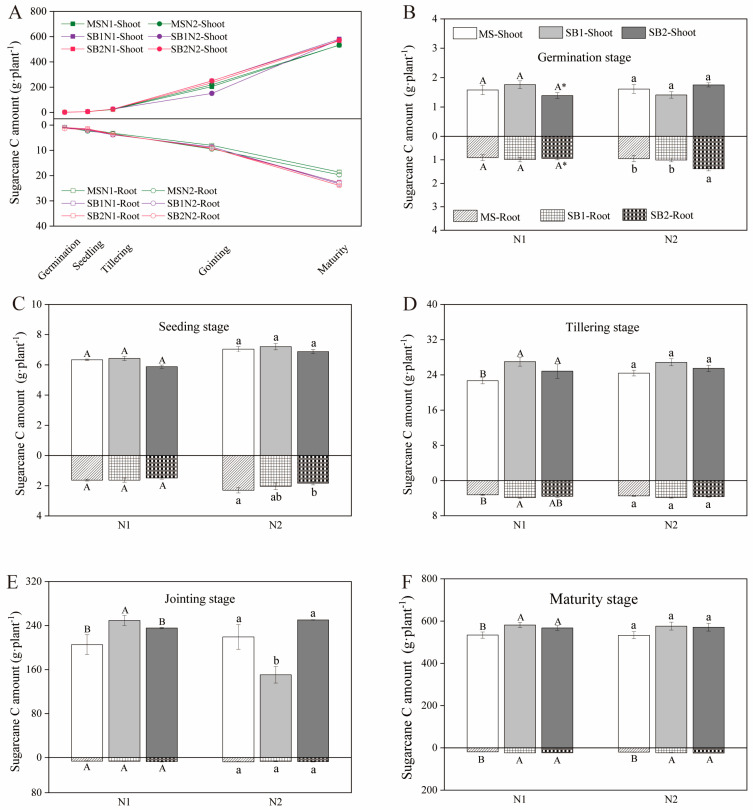
Changes in shoot and root C amount of sugarcane during a growth period in 2022 (**A**). Effects of cropping pattern and N application on the shoot and root C amount of sugarcane at germination stage (**B**), seeding stage (**C**), tillering stage (**D**), jointing stage (**E**), and maturity stage (**F**). Different uppercase letters indicate significant differences between different cropping patterns at the N1 level, different lowercase letters indicate significant differences between different cropping patterns at the N2 level, and * indicates significant differences between N1 and N2 in the same cropping pattern.

With the growth and development of sugarcane, the shoot and root C sequestration of sugarcane showed an upward trend (Figure 1 and Table S1). From the germination stage to the tillering stage of sugarcane, the growth rate of plant C sequestration was relatively slow. After entering the tillering stage of sugarcane, the growth rate of plant C sequestration accelerated significantly (Figure 1A). At the sugarcane germination stage, the root C sequestration of SB2 was 45.26% and 36.63% higher than that of MS and SB1, respectively, at the N2 level (Figure 1B). At the tillering stage of sugarcane, the shoot C sequestration of the intercropping pattern was significantly higher than that of the monocropping pattern at the N1 level, and the root C sequestration of SB1 was the highest and significantly higher than in MS (Figure 1D). The planting pattern only affected the aboveground C sequestration at the jointing stage of rapid growth of sugarcane (Figure 1E). At the mature harvest of sugarcane, the shoot and root C sequestration of the intercropping was significantly higher than that of MS, except that the increase of shoot C sequestration under the N2 level was not significant (Figure 1E and Table S1). These results indicate that sugarcane/soybean intercropping and reduced N application can increase the C sequestration of sugarcane aboveground and in the roots during the middle and late stages of sugarcane growth.

### 2.2. Dynamic Changes of C Sequestration in the Shoots and Roots of Soybean

**Figure 2:**
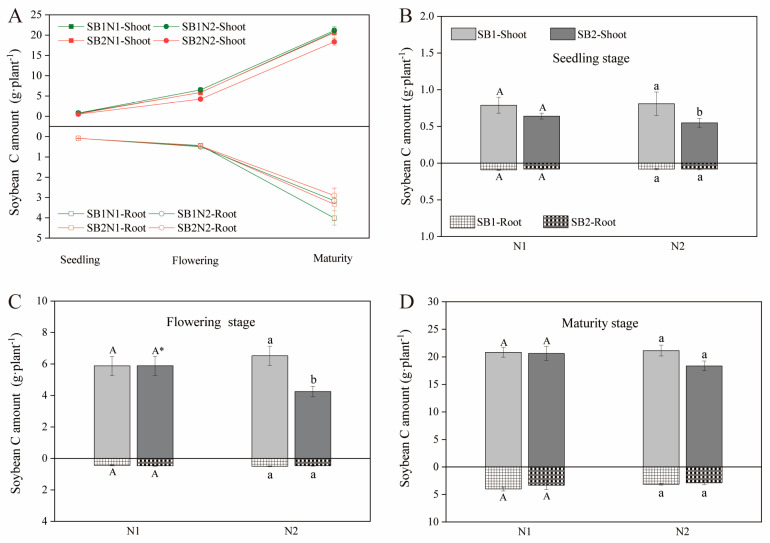
Changes in shoot and root C amount of soybean during a growth period in 2022 (**A**). Effects of cropping pattern and N application on the shoot and root C amount of soybean at seeding stage (**B**), flowering stage (**C**), and maturity stage (**D**). Different uppercase letters indicate significant differences between different cropping patterns at the N1 level, different lowercase letters indicate significant differences between different cropping patterns at the N2 level, and * indicates significant differences between N1 and N2 in the same cropping pattern.

The C sequestration of soybean shoots and roots increased with the growth period of soybean (Figure 2 and Table S2). The growth rate of C sequestration in soybean seedlings is relatively slow. After the soybean flowering period, the growth rate increased significantly, i.e., the growth rates of shoots and roots were 0.67–0.72 g/plant/day and 0.12–0.17 g/plant/day, respectively (Figure 2A). The shoots and roots C content of soybean were not lower than those of conventional N application at reduced N application levels during flowering, seedling, and maturity (Figure 2B,C and Table S2). These results indicate that reducing N application in sugarcane/soybean intercropping patterns can maintain the growth and development of soybeans without reducing the C sequestration of the shoots and roots.

### 2.3. Dynamic Changes of C Sequestration in the Shoots and Roots of the System

**Figure 3:**
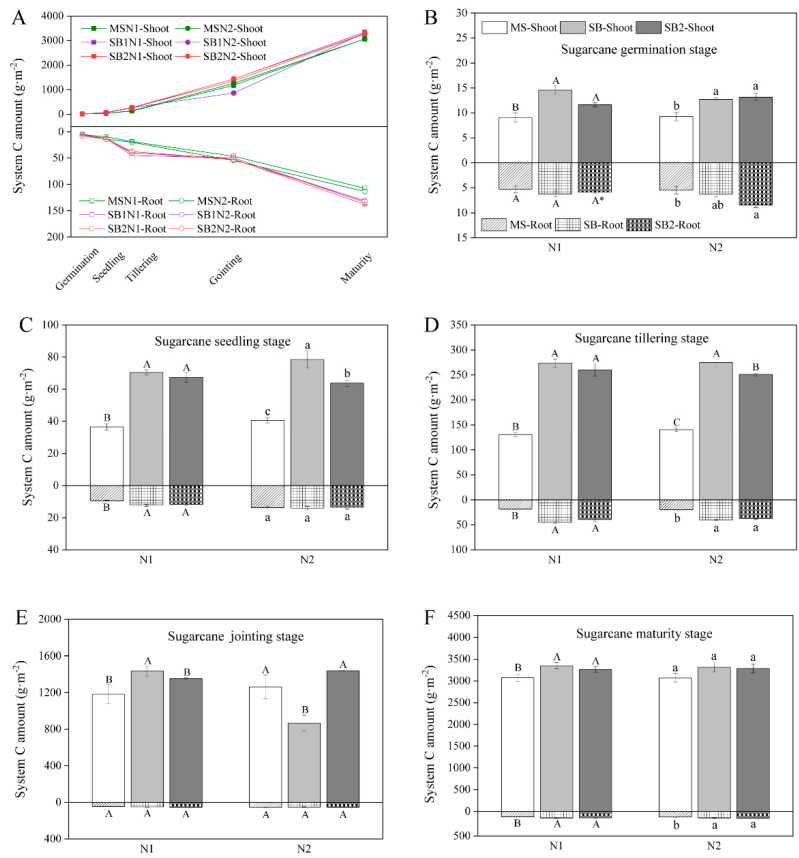
Changes in shoot and root C amount of the system during the sugarcane growth period in 2022 (**A**). Effects of cropping pattern and N application on the shoot and root C amount of the system at sugarcane germination stage (**B**), sugarcane seeding stage (**C**), sugarcane tillering stage (**D**), sugarcane jointing stage (**E**), and sugarcane maturity stage (**F**). Different uppercase letters indicate significant differences between different cropping patterns at the N1 level, different lowercase letters indicate significant differences between different cropping patterns at the N2 level, and * indicates significant differences between N1 and N2 in the same cropping pattern.

The amount of C sequestration in the shoots and roots of the system increased with the growth and development of sugarcane and soybean (Figure 3 and Table S3). The growth pattern of systemic C sequestration was similar to that of sugarcane, where C sequestration by shoots and roots increased by 38.7–80.6 and 6.7–9.3 times, respectively, at maturity compared with the tillering stage (Figure 3A). At the germination stage of sugarcane, the shoot C sequestration in the intercropping pattern was significantly higher than that in the MS at the N1 and N2 levels (Figure 3B and Table S3). At the seedling stage of sugarcane, the effect of intercropping mode on the system C sequestration was more significant. The shoot and root C sequestration at the N1 level and shoot C sequestration at the N2 level in intercropping mode were significantly higher than those in MS, especially in the SB1 intercropping pattern (Figure 3C and Table S3). When sugarcane is in the jointing stage of rapid growth, the planting pattern only affects the shoot C sequestration in the system. In particular, the shoot C sequestration in the system of intercropping patterns at the N1 level was significantly higher than that of the MS, especially the SB1 intercropping pattern (Figure 3E). At sugarcane maturity and harvest, the intercropping pattern significantly increased the shoot and root C sequestration.

### 2.4. Dynamic Changes of Soil TOC Content

**Figure 4:**
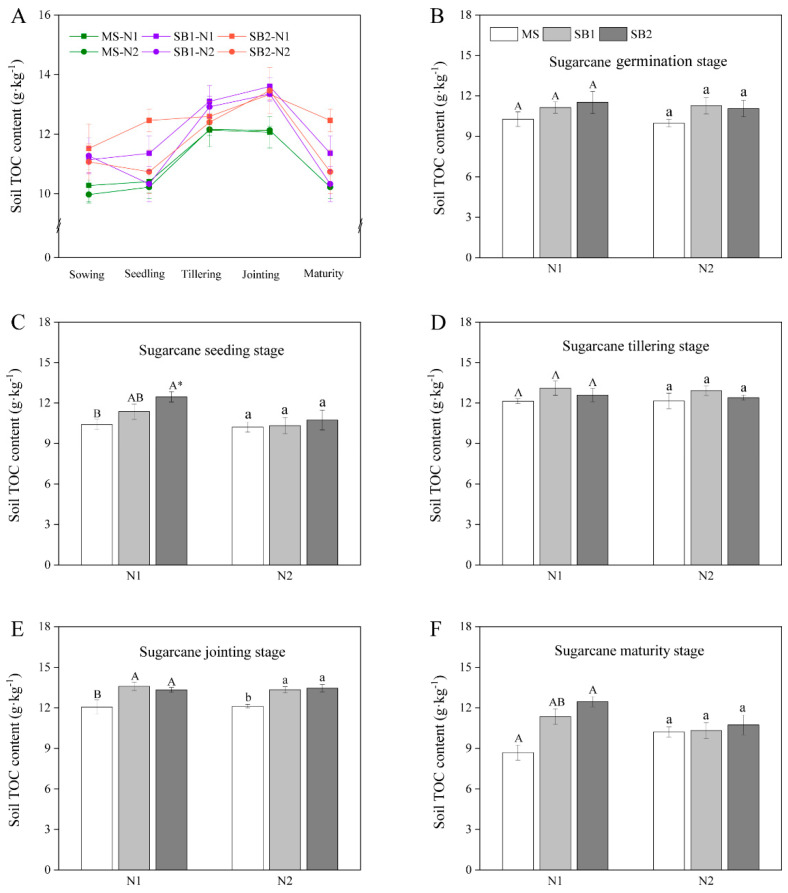
Changes in soil TOC content during the sugarcane growth period in 2022 (**A**). Effects of cropping pattern and N application on the soil TOC content at sugarcane germination stage (**B**), sugarcane seeding stage (**C**), sugarcane tillering stage (**D**), sugarcane jointing stage (**E**), and sugarcane maturity stage (**F**). Different uppercase letters indicate significant differences between different cropping patterns at the N1 level, different lowercase letters indicate significant differences between different cropping patterns at the N2 level, and * indicates significant differences between N1 and N2 in the same cropping pattern.

Soil TOC content increased and then decreased during the growth period of sugarcane and reached a maximum at the jointing stage (Figure 4A and Table S4). At the seedling stage, the soil TOC content at the N1 level was SB2 > SB1 > MS, and the soil TOC content of SB2 was significantly higher than that of MS (Figure 4C). When sugarcane was at the jointing stage, the effect of the planting pattern on soil TOC was more obvious; that is, the soil TOC content in SB1 and SB2 was significantly higher than that in MS under N1 application levels (Figure 4E and Table S4). At the maturity stage, SB2 and SB1 were 30.65% and 43.33% higher than the MS at the N1 level, respectively (Figure 4F). In addition, there was no significant difference in soil TOC content between different N levels in the same planting pattern at each growth stage of sugarcane, except for the seedling stage of SB2. These results indicated that sugarcane/soybean intercropping and reduced N application promoted the increase of soil TOC content, which was conducive to the maintenance of soil fertility.

### 2.5. Dynamics Changes of Soil MBC Content

**Figure 5:**
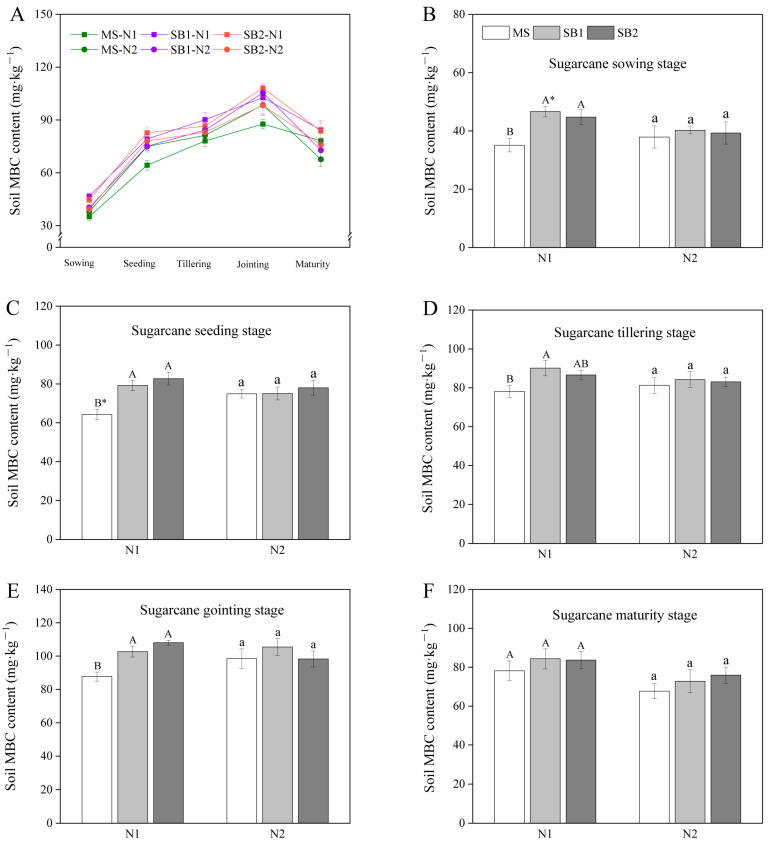
Changes in soil MBC content during a sugarcane growth period in 2022 (**A**). Effects of cropping pattern and N application on the soil MBC content at sugarcane germination stage (**B**), sugarcane seeding stage (**C**), sugarcane tillering stage (**D**), sugarcane jointing stage (**E**) and sugarcane maturity stage (**F**). Different uppercase letters indicate significant differences between different cropping patterns at the N1 level, different lowercase letters indicate significant differences between different cropping patterns at the N2 level, and * indicates significant differences between N1 and N2 in the same cropping pattern.

Soil MBC, as an active organic C component, is more sensitive to changes in external environmental factors. The soil MBC content increased continuously from before germination to the jointing stage, and the content was the highest at the jointing stage (Figure 5A and Table S5). The MBC content in SB1 and SB2 at the N1 level was significantly higher than that in MS before germination and at the tillering stage (Figure 5B,D and Table S5). At the seedling stage, the MBC content at the N1 level was SB2 > SB1 > MS, and SB2 was significantly higher than MS (Figure 5C). At the jointing stage, the planting pattern only affected the MBC content at the N1 level; that is, the soil microbial biomass C content of SB1 and SB2 was significantly higher than that of MS (Figure 5E and Table S5). At the maturity stage, the MBC content of SB1 and SB2 was 7.48% and 9.8% higher than that in MS at the N1 level, respectively (Figure 5F). In addition, there was no significant difference in MBC content between different N levels in the same planting pattern at different growth stages of sugarcane, except for the sowing stage of SB1. These results indicated that both sugarcane/soybean intercropping and reduced N application can significantly increase soil MBC content throughout sugarcane growth.

### 2.6. Dynamic Changes of Soil DOC Content

**Figure 6:**
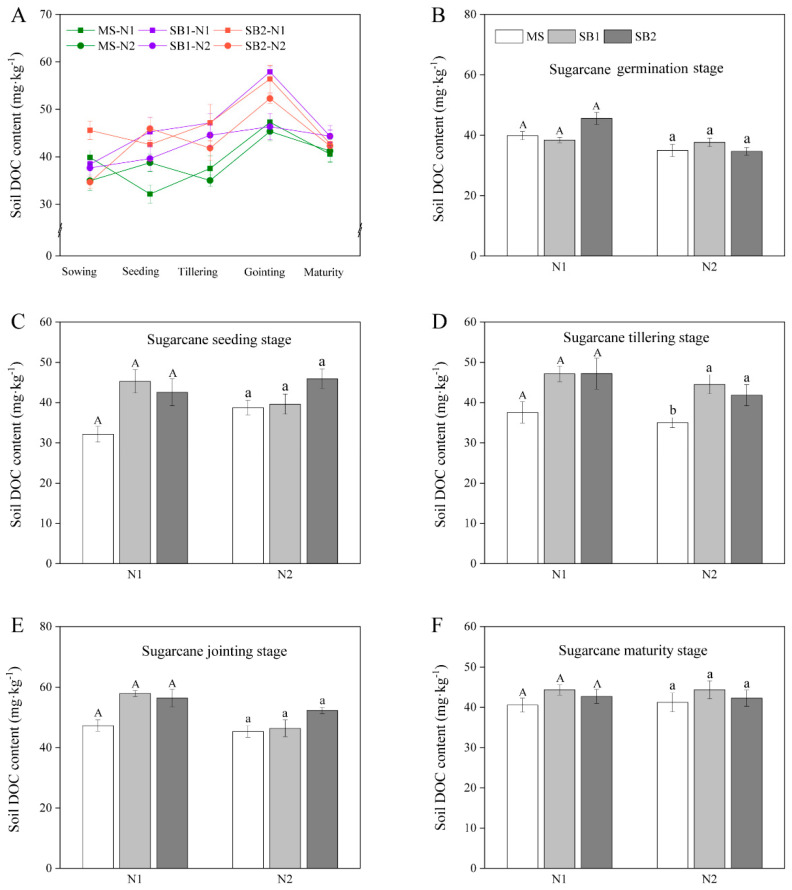
Changes in soil DOC content during a sugarcane growth period in 2022 (**A**). Effects of cropping pattern and N application on the soil DOC content at sugarcane germination stage (**B**), sugarcane seeding stage (**C**), sugarcane tillering stage (**D**), sugarcane jointing stage (**E**), and sugarcane maturity stage (**F**). Different uppercase letters indicate significant differences between different cropping patterns at the N1 level, different lowercase letters indicate significant differences between different cropping patterns at the N2 level.

The soil DOC content showed a significant fluctuation change due to the influence of external environmental factors. The soil DOC content increased first and then decreased during the whole sugarcane growth period and reached the highest at the jointing stage (Figure 6A and Table S6). At the seedling stage, the soil DOC content of SB1 and SB2 at the N1 level was 40.81% and 32.41% higher than that of MS, respectively (Figure 6C). For the tillering stage, the soil DOC content of SB1 at the N2 level was the highest and significantly higher than that in MS (Figure 6D). At the jointing stage, the planting pattern only affected the soil DOC content at the N1 level (Figure 6E). At the maturity stage, the soil DOC content of SB1 and SB2 at the N1 level was 7.3% and 5.04% higher than that of MS, respectively, but did not reach a significant level (Figure 6F). In addition, there was no significant difference in soil DOC content at different N levels in the same planting pattern at different growth stages of sugarcane. These results indicated that sugarcane/soybean intercropping and reduced N application promoted the increase of soil DOC content (Figure 6 and Table S6).

### 2.7. Regression Analysis of Plant C Sequestration and Soil Organic C Components

**Figure 7:**
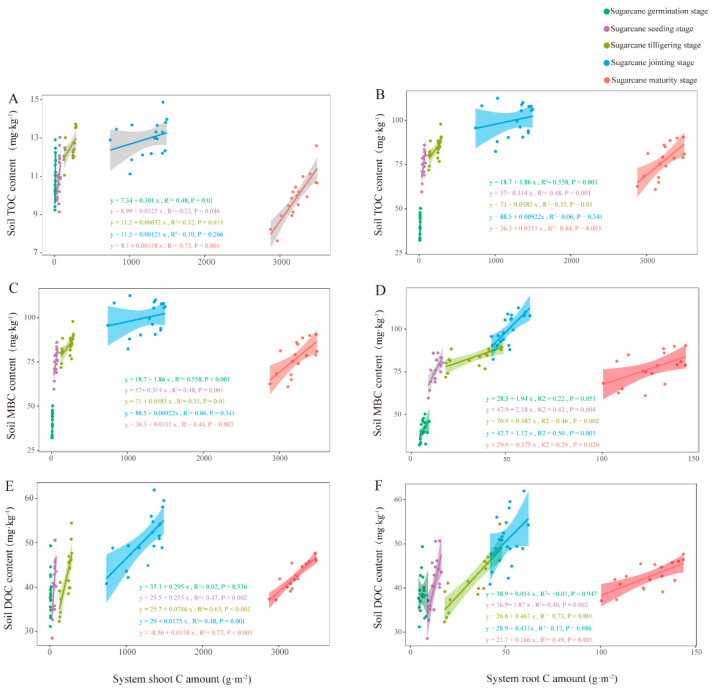
Regression analysis of system shoot and root C amount with soil TOC (**A**,**B**), MBC (**C**,**D**), and DOC (**E**,**F**) content.

Regression analyses were carried out on the content of organic C components in the shoot, root, and soil of the system. There was a significant regression relationship (*p* < 0.05) between the shoot and root C sequestration in the system and the soil TOC content at all growth periods of sugarcane, except for the jointing stage (Figure 7A,B). As for soil MBC content, it had a significant regression relationship (*p* < 0.05) with the shoot and root C sequestration in the system at all growth periods of sugarcane, except for the C sequestration of the upper part of the jointing stage (Figure 7C,D). Different from TOC and MBC, there was no significant regression relationship between soil DOC content and shoot and root C sequestration in the system at the germination stage of sugarcane. However, there were significant regressions between soil DOC content and root and shoot C sequestration in the system during the rest of the sugarcane growth period (Figure 7E,F).

### 2.8. Effects of Planting Pattern and N Application on Soil Nutrients and Sugarcane Yield

**Figure 8:**
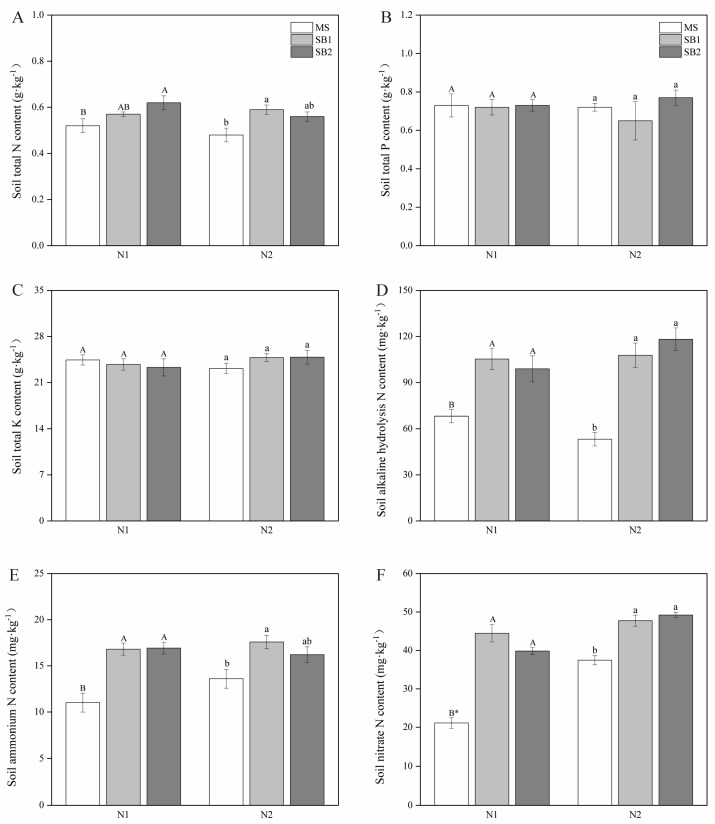
Effects of planting pattern and N application rate on soil nutrient content in 2022. Different uppercase letters indicate significant (*p* < 0.05) differences between different cropping patterns at the N1 level, different lowercase letters indicate significant (*p* < 0.05) differences between different cropping patterns at the N2 level, and * indicates significant (*p* < 0.05) differences between N1 and N2 in the same cropping pattern. Soil total N content (**A**), soil total P content (**B**), soil total K content (**C**), soil alkaline hydrolysis N content (**D**), soil ammonium N content (**E**) and soil nitrate N content (**F**).

**Figure 9:**
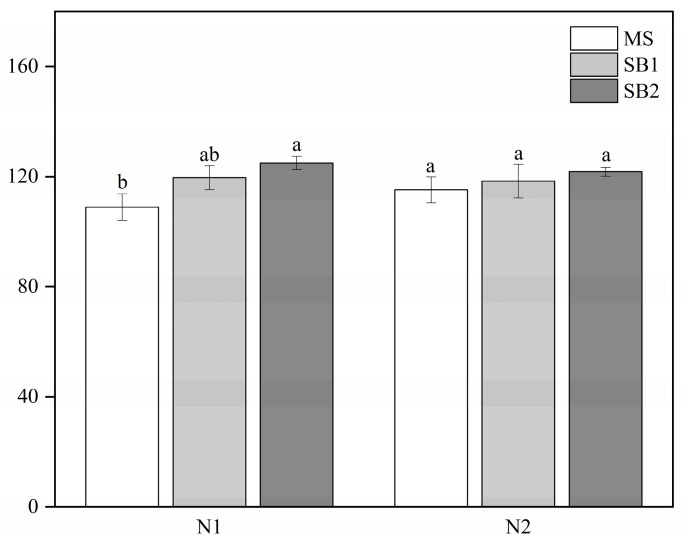
Effects of planting pattern and N application rate on sugarcane yield in 2022. Different lowercase letters indicate significant (*p* < 0.05) differences between different cropping patterns at the N2 level.

As shown in Figure 8A, sugarcane/soybean intercropping significantly affected the total N content of the soil, which was significantly higher in the SB2 treatment than in the MS treatment at the N1 level, whereas the total N content of the soil was significantly higher in both the SB2 and SB1 treatments than in the MS treatment at the N2 level. Planting pattern and N application did not significantly affect the total P and total K content of the soil (Figure 8B,C and Table S7). Both sugarcane/soybean intercropping patterns significantly increased alkaline hydrolysis N, nitrate nitrogen, and ammonium nitrogen contents in the soil compared to sugarcane monocropping, whereas the amount of N applied only affected soil nitrate N content at the N1 level (Figure 8D–F and Table S7). Sugarcane yield was not reduced in the sugarcane/soybean intercropping pattern and was also significantly higher in the SB2 treatment than in the MS treatment at the N1 level (Figure 9). In 2021, sugarcane yield was significantly higher in the sugarcane/soybean intercropping pattern than in the sugarcane monoculture pattern at both N application levels (Figure S1). These results suggest that sugarcane/soybean intercropping can improve soil nutrient status and maintain or increase sugarcane yield.

## 3. Discussion

### 3.1. Plant C Sequestration in the Sugarcane/Soybean Intercropping System

The C fixed by crops through photosynthesis is an important C input in agroecosystems and one of the main sources of SOC [25]. Intercropping can fully utilize light, heat, water, nutrients, and land resources [26] and can also use the symbiotic relationship between species to improve land use efficiency and crop yield [27]. Multiple intercropping patterns have been shown to improve crop yields, among which gramineous/leguminous intercropping is more conducive to the increase of crop yield. Examples include wheat (*Triticum aestivum* L.)/soybean [28], maize/soybean [29], and sugarcane/soybean [30]. Among various intercropping patterns, gramineous/leguminous intercropping is the most favorable for increasing crop yield [31]. Yield advantage is based on the biomass accumulation and distribution of absorbed nutrients to the reproductive organs [32]. Gramineae/leguminosae intercropping is more favorable for the accumulation of plant biomass than the monocropping pattern. For example, barley/soybean intercropping and alfalfa/maize intercropping significantly increased the total biomass of plants compared to maize monocropping [23,24]. In agroecosystems, the greater the plant biomass per unit area of soil, the greater the C sequestration of plants. Therefore, more photosynthetic C can be released into the soil. Our study found that the total shoot and root C sequestration in the intercropping system was significantly higher than that in the monocropping system during the whole growth period of sugarcane, which was similar to the previous results on biomass accumulation in the intercropping system. The complementarity of resources and niches between different plants in the gramineous/leguminous intercropping system promoted the growth and development of plants and increased plant C sequestration. The complementarity of the niche is categorized into aboveground complementarity and belowground complementarity. For example, in the maize/faba bean (*Vicia faba* L.) intercropping system, the faba bean roots are shallow and distributed in the upper layer of the soil, which allows the maize roots to extend below the faba bean roots to explore a larger soil range to obtain nutrients and water [33]. The niche complementarity of the aboveground part is mainly to increase the interception of light by the crop. Yu et al. [34] showed in a meta-analysis that the high light interception rate of the intercropping system was related to niche differentiation. Zhou et al. [35] also found that the light capture in the maize/soybean intercropping system was higher than that in soybean monoculture.

The effect of the cropping pattern on the C sequestration of sugarcane varied with the N application rate, i.e., intercropping significantly increased the C sequestration of shoots and roots compared with sugarcane monocropping under the reduced N application level (300 kg·hm^−2^). However, there was no significant difference in plant C sequestration between different planting patterns at the conventional N application level (525 kg·hm^−2^). It may be attributed to the fact that sugarcane is a crop with high N demand, and the N application rate of 300 kg·hm^−2^ cannot meet its growth and development needs. When sugarcane is intercropped with soybeans, sugarcane can utilize the N fixed by soybeans to supplement the N demand and increase its own C sequestration [36]. As an N-fixing crop, soybeans can fix a large amount of N from the air through biological N fixation, so excessive chemical N fertilizer in the soil may inhibit its growth and development [37]. The results of this experiment proved this view that the increase of N application in SB2 significantly reduced the shoot C sequestration of soybeans. In addition, we also found that the increase of N application level in the intercropping system did not increase the C sequestration of the sugarcane shoot and root, which indicated that the N application level of 300 kg·hm^−2^ in the sugarcane/soybean intercropping system could meet the N demand of sugarcane growth and development. By studying N transfer in the sugarcane/soybean intercropping system, Zhang et al. [36] found that the reason why sugarcane can maintain plant biomass at a low N level in the intercropping system is mainly due to the transfer of N from soybean to sugarcane. Soybean transfers N to adjacent sugarcane plants mainly through roots, arbuscular mycorrhizal hyphae (AMF), and root exudates, thus reducing the demand for chemical N fertilizer in sugarcane.

### 3.2. Soil TOC Fixation in the Sugarcane/Soybean Intercropping System

Plant rhizosphere sediments are an important source of soil organic C, and their quantity and quality are controlled by plant growth and development. The distribution of plant biomass directly drives rhizosphere deposition. In this study, sugarcane/soybean intercropping increased the shoot and root C sequestration of the system, which means that more rhizosphere sediments may be released into the soil in the sugarcane/soybean intercropping system, thus promoting the increase of soil organic C content. The view that intercropping patterns can increase SOC content has been confirmed by many studies. Hu et al. [21] showed that SOC content in sugarcane/soybean and sugarcane/grass pea (*Mucuna pruriens* var. *utilis*) intercropping systems were significantly higher than that in sugarcane monocropping. Compared with maize monocropping, maize/cowpea (*Vigna unguiculata* L.) intercropping had the highest SOC sequestration of 0.17 mg C·ha^−1^·yr^−1^ in low fertility soil [38]. This study found that the SOC of the intercropping system was generally higher than that of the sugarcane monocropping system during the growth period of sugarcane, which was similar to the results of previous studies. It should be pointed out that the effect of intercropping on SOC content is more prominent at the N1 level, which is the most advantageous N application level in sugarcane/soybean intercropping field practice [30].

Crop residue return is also the reason for the increase of SOC content in the sugarcane/soybean intercropping system. The photosynthetic C input into the soil through crop residues was much higher than that in rhizosphere sediments. In rice (*Oryza sativa* L.), only 10.8% of the photosynthetic C was transferred to the soil [39]. Jin et al. [40] also pointed out that soybeans only distributed 2.1% of net assimilated C to the soil in the form of rhizosphere sediments at maturity. Therefore, crop residue is important for the increase of soil organic C content. In the conventional sugarcane monocropping pattern, the low organic C input in the sugarcane field is because sugarcane needs to be recycled as a sugar crop stem, and only leaves can be returned to the soil. In the sugarcane/soybean intercropping system, soybean straw can be returned to the soil after soybean harvest, which is more conducive to the increase of soil organic C content. In addition, Lian et al. [41] also showed that sugarcane/soybean intercropping changed soil microbial function and improved the C sequestration capacity of prokaryotes in soil.

### 3.3. Fixation of Soil Labile Organic C in the Sugarcane/Soybean Intercropping System

MBC is sensitive to agricultural management and is often used as an early indicator of change in nutrient cycling and dynamic changes in soil organic matter, which can accurately reflect soil microbial activity and environmental quality [42,43]. Diversified ecological agricultural cropping systems affect soil microbial habitats and thus change their community structure characteristics [44]. Silva et al. [45] found that forage/legume intercropping can improve microbial activity in grassland ecosystems. Compared with single forage planting, the soil MBC in forage/cowpea and forage/chickpea (*Cicer arietinum* Linn.) intercropping systems increased significantly. In the agroecosystems, the intercropping of cereal crops and legume crops also promoted the growth of microorganisms. The MBC content in the rhizosphere soil of wheat/chickpea and wheat/lentil (*Lablab purpureus* L.) intercropping systems was significantly higher than that of wheat monocropping [46]. Similar to the results of previous studies, this study found that the soil MBC content of sugarcane/soybean intercropping at the N1 level was significantly higher than that of MS at the sugarcane germination stage, seedling stage, tillering stage, and jointing stage. The intercropping system is characterized by a higher diversity of roots and residues, which improves the energy supply of soil microbial biomass by releasing exudates such as amino acids and organic acids from the roots [47]. Thus, the effects of these residues on soil microbial biomass were more pronounced in intercropping treatments than in monocropping treatments. Kooch et al. [48] also observed that intercropping can increase the number and diversity of soil microorganisms by increasing more diverse C sources for microorganisms in soil due to its plant diversity. In addition, intercropping patterns can also regulate microbial communities by affecting soil physical and chemical properties [49]. Soil physicochemical properties are the key factors regulating soil microbial activities. Based on the composition and chemical properties of the soil matrix and nutrient utilization efficiency, the composition and diversity of soil microbial communities were also affected [50]. Soil pH has always been considered to be the main factor determining soil microbial activity and community composition [51,52]. Some researchers also believe that soil NH_4_^+^-N and NO_3_^−^-N are the key factors [50]. In this study, the contents of NH_4_^+^-N and NO_3_^−^-N in the soil of sugarcane/soybean intercropping at reduced N application were significantly higher than those in sugarcane monoculture and had a significant correlation with MBC content in the soil.

As the most active component of organic C, soil DOC is the main form of organic C migration and transformation. It is easily utilized by soil microorganisms to convert into more stable organic matter stored in the soil or decomposed into CO_2_ released into the atmosphere [53]. The study showed that the soil DOC content in the sugarcane/soybean intercropping system was generally higher than that in the sugarcane monocropping during the growth period of sugarcane. It may involve the following factors. First, the organic matter returned to the soil after intercropping increased, thereby expanding the substrate. In this study, the aboveground straw of monocropping and intercropping sugarcane was removed from the soil. The sugarcane and soybean roots, litter, rhizosphere sediments, and soybean straw that naturally fell off before harvest were returned to the soil. Therefore, we speculated that the main reason for the higher soil DOC content after intercropping than monocropping was more input of organic matter. In addition, intercropping is a diversified planting pattern that alters the physicochemical properties of soil, such as temperature and humidity [54] and provides a favorable soil environment for microbial activities. Compared with monocropping maize, soil temperature increased by 1.61 °C after maize/potato (*Solanum tuberosum* L.) intercropping, and DOC content had a significant linear relationship with temperature [55]. It may be attributed to the fact that elevated temperature increases the activity of microorganisms, accelerates the utilization of plant residues, and ultimately promotes the production of DOC [56]. The stems and leaves of intercropping soybeans naturally withered, and the litter entered the soil, which strengthened the diversity and continuity of organic matter input. Previous studies have mentioned that diversified planting enriches the source of organic matter, thereby increasing soil microbial activity and diversity and ultimately increasing DOC content [54]. The continuity of organic matter input means that soybean roots, stems, and leaves enter the soil at an early stage, and maize roots and litter return to the soil later. Therefore, the activity of the soil microbial community to decompose organic C will continue to be maintained at a high level, eventually leading to the accumulation of DOC [57].

## 4. Materials and Methods

### 4.1. Field Sites

The experiment was conducted from March 2009 to December 2022 at the Experimental Center of South China Agricultural University (23°08′ N, 113°15′ E). All experimental designs and field management were consistent, and the results of plant and soil C sequestration were observed in 2021 and 2022. The area was located in the subtropics and has a typical subtropical monsoon oceanic climate, with sufficient light and heat resources, annual sunshine hours of 1272.9–2147.9 h, total solar radiation of 105.3 kJ·cm^−2^, average temperature of 21.9–22.8 °C, average rainfall of 1384–2278 mm, and about 85% of the precipitation is concentrated in the months of April–September. The soil in the experimental field was a lateritic red soil. The initial topsoil contained organic matter 21.08 g·kg^−1^, alkali-hydrolyzable N 75.38 mg·kg^−1^, available phosphorus 75.04 g·kg^−1^, and available potassium 61.71 g·kg^−1^.

### 4.2. Experimental Materials

The variety of sugarcane used in the experiment was Yuetang 00-236 provided by the farm of South China Agricultural University, which has the characteristics of early maturity, high sugar, high yield, fast and neat germination, high germination rate, strong tillering ability, and high stem rate. The soybean variety used in the experiment was the early-maturing soybean variety Maodou NO. 3, with a growth period of about 100 days, which was provided by the Guangdong Branch of the National Soybean Improvement Center.

### 4.3. Experiment Design

A two-factor, completely randomized block design (cropping pattern and N application level) was used to conduct a long-term experiment, with a total of 7 treatments (Table 1). The cropping patterns included sugarcane monocropping (MS), sugarcane/soybean 1:1 intercropping (SB1), and sugarcane/soybean (1:2) intercropping (SB2). The two N application levels were reduced N application level (300 kg·hm^−2^) and conventional N application level (525 kg·hm^−2^). In addition, a non-fertilized soybean monocropping control (MB) was set. Each treatment was repeated three times, and each plot was 26.4 m^2^ (5.5 m × 4.8 m). The row spacing of sugarcane was 1.2 m, 4 rows were planted in each plot, and 38 double-bud seedlings were planted in each row. In each plot, the row spacing of soybean was 0.3 m, the plant spacing was 0.2 m, 25 holes were planted in each row, and 2 plants were planted in each hole at the seedling stage. Four rows, eight rows, and sixteen rows of soybeans were planted in SB1, SB2, and MB, respectively.

**Figure 10:**
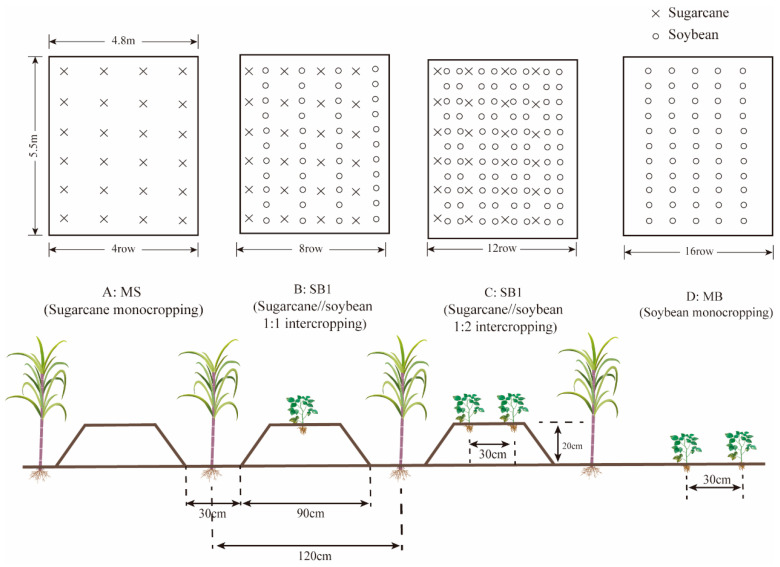
Diagram of sugarcane and soybean field planting.

From 2009 to 2022, sugarcane is sown in late February or early March according to weather conditions, and soybean is sown after 7 days of sugarcane planting. Soybeans are harvested in late May or early June, and sugarcane in mid-to-late December. When planted each year, the position of sugarcane and soybean is fixed, and soybean leaves and stems are returned to sugarcane rows. Sugarcane/soybean intercropping adopts the furrow mode. Soybean was planted in the furrow of 90 cm width, and sugarcane was planted in the furrow of 30 cm width. The planting details are shown in Figure 10. Sugarcane basal fertilizer is applied in the ditch where sugarcane is planted, and the fine soil after 5 cm is covered. The follow-up fertilizer is applied in the ditch of sugarcane planting, and then the soil is cultivated. Soybean is not fertilized during the whole growth period. In the intercropping pattern, the leaves and stems of soybean were returned to the sugarcane row after harvest, and the soil was covered. The border originally planted with soybeans becomes a ditch, which is conducive to drainage. Other field management is consistent with local sugarcane planting.

### 4.4. Soil and Plant Sampling Collection

In 2021 and 2022, sugarcane and soybean field plants were sampled at the sugarcane germination stage (soybean seedling stage), seedling stage (soybean flowering stage), tillering stage (soybean maturity stage), jointing stage, and maturity stage, respectively. The shoot and root parts of 3 representative sugarcane and 3 soybean plants were selected from each plot separately. The samples were dried, and the dry matter weight was measured and then crushed through a 100-mesh sieve to determine the C content of each organ. At the same time, 5 samples of the 0–30 cm soil layer were sampled by the five-point method in the middle position of the sugarcane planting row and soybean planting row before the sowing, seedling stage, tillering stage, elongation stage, and maturity stage of sugarcane, respectively. The samples were divided into two parts. One part was stored in a refrigerator at 4 °C for the determination of MBC and DOC, and the other part was used for the determination of TOC after natural air drying.

### 4.5. Contents of TOC, MBC, and DOC in Soil

The MBC in fresh soil was determined by chloroform fumigation extraction [58]. The fresh soil equivalent to 10 g dried was fumigated for 24 h, then 40 mL 0.5 mol·L^−1^ K_2_SO_4_ was added and oscillated at 250 r/min for 1 h. Finally, the test solution was obtained by filtration with a 0.45 μm filter membrane. The same amount of soil was also extracted without fumigation for the determination of DOC in the soil. MBC was calculated based on the difference in total organic C content between the fumigated and unfumigated soil extracts and corrected using the conversion coefficient (kEC) value of 0.45. The content of organic C in soil extract was measured by Element high TOC II (Burladingen, Germany). Soil TOC was determined using the potassium dichromate volumetric method.

### 4.6. Calculation Method

According to the following formula, the amount of C sequestration in the shoots and roots of a single plant was calculated:

P_cs_ = P_b_ × P_cc_ (1)

P_cs_ refers to the C sequestration of each part of the plant (g), P_b_ refers to the biomass of each part of the plant (g), and P_cc_ refers to the C content of each part of the plant (%). The C sequestration of the system (g·m^−2^) is the C sequestration of plants per unit area of land.

### 4.7. Statistical Analysis

SPSS 22.0 (SPSS, Chicago, IL, USA) and Microsoft Excel 2010 were used for data processing and statistical analysis. The Duncan multiple comparison method was used to test the significant difference (*p* < 0.05). Linear regression analysis was used to evaluate the relationship between plant C sequestration and soil organic C components. Origin 2020 (Origin Lab Corporation, Northampton, MA, USA) was adopted for plotting.

## 5. Conclusions

The results of this study showed that sugarcane/soybean intercropping increased the C sequestration of shoots and roots of sugarcane compared with MS treatment during the whole growth period of sugarcane. Consistent with the change rule of plant C sequestration, soil labile organic C content and SOC content were higher in the sugarcane/soybean intercropping pattern, and this increased effect was more obvious under the condition of reduced N application. Regression analysis showed that shoot and root C sequestration were significantly correlated with SOC content. These results suggested that the sugarcane/soybean intercropping may increase the input of exogenous organic C in the soil by increasing the C sequestration of the plant, thereby promoting SOC sequestration.

## Figures and Tables

**Table 1:** Field experiment design of cropping pattern and nitrogen rate application.

Treatment	Nitrogen Application	Cropping Patterns
MSN1	300	Sugarcane monocropping
SB1N1	300	Sugarcane/soybean intercropping (1:1)
SB2N1	300	Sugarcane/soybean intercropping (1:2)
MSN2	525	Sugarcane monocropping
SB1N2	525	Sugarcane/soybean intercropping (1:1)
SB2N2	525	Sugarcane/soybean intercropping (1:2)
MB	0	Soybean monocropping

## Data Availability

The dataset is available upon request from the authors. The data are not publicly available due to [the data might be used in ongoing or future studies. To preserve the integrity and originality of forthcoming research, the data is kept confidential until these studies are completed].

## Supplementary Materials

The following supporting information can be downloaded at: https://www.mdpi.com/article/10.3390/plants13162337/s1, Table S1. Effects of planting pattern and N application rate on the shoot and root C amount (g·m^−2^) of sugarcane at different growth stages in 2021; Table S2. Effects of planting pattern and N application rate on the shoot and root C amount (g·m^−2^) of soybean at different stages in 2021; Table S3. Effects of planting pattern and N application rate on the shoot and root C amount (g·m^−2^) of system at different sugarcane growth stages in 2021; Table S4. Effects of planting pattern and N application rate on the soil TOC content (g·kg^−1^) at different sugarcane growth stages in 2021; Table S5. Effects of planting pattern and N application rate on the soil MBC content (mg·kg^−1^) at different sugarcane growth stages in 2021; Table S6. Effects of planting pattern and N application rate on the soil DOC content (mg·kg^−1^) at different sugarcane growth stages in 2021; Table S7. Effects of planting pattern and N application rate on the soil nutrient content in 2021; Figure S1. Effects of planting pattern and N application rate on sugarcane yield in 2021.
