# Supplementary material for: Sugarcane/Soybean Intercropping with Reduced Nitrogen Application Synergistically Increases Plant Carbon Fixation and Soil Organic Carbon Sequestration
Source: Plants (Basel). 2024 Aug 22;13(16):2337. doi: 10.3390/plants13162337 (PMC11359578; doi:10.3390/plants13162337)
Supplement: Supplementary file 1 [file plants-13-02337-s001.zip › plants-3142885-supplementary.pdf]

Sugarcane/Soybean Intercropping with Reduced Nitrogen Application Synergistically Increases Plant Carbon Fixation and Soil Organic Carbon Sequestration

Tantan Zhang [1,2,*], Yali Liu [3] and Lin Li [4,*]

[1] College of Chemistry and Bioengineering, Hunan University of Science and Engineering, Yongzhou 425199, China
[2] Hunan Engineering Technology Research Center for Comprehensive Development and Utilization of Biomass Resources, Yongzhou 425199, China
[3] College of Natural Resources and Environmental, South China Agricultural University, Guangzhou 510642, China
[4] College of Breeding and Multiplication (Sanya Institute of Breeding and Multiplication), Hainan University, Sanya 572000, China
* Correspondence: 13610205677@163.com (T.Z.); lilin18@hainanu.edu.cn (L.L.)

Table S1. Effects of planting pattern and N application rate on the shoot and root C amount ($g{\cdot}m^{-2}$) of sugarcane at different growth stages in 2021

		Sugarcane germination stage	sugarcane seeding stage	sugarcane tillering stage	sugarcane jointing stage	sugarcane maturity stage
Shoot	MS-N1	1.89±0.19a	6.68±0.25b	24.61±0.99b	187.62±13.9a	486.88±13.24b
	SB1-N1	2.11±0.15a	8.64±0.34ab	30.06±0.90a	228.53±7.16a	525.86±7.93a
	SB2-N1	1.84±0.08a	9.72±1.18a	30.67±1.88a	232.23±5.68a	529.83±9.88a
	MS-N2	1.93±0.17a	7.98±0.18a	26.51±1.05a	207.31±17.87a	504.98±8.32a
	SB1-N2	2.03±0.11a	8.33±0.40a	29.21±1.19a	233.34±19.32a	534.23±8.19a
	SB2-N2	2.20±0.18a	7.79±0.33a	28.37±0.84a	232.66±0.40a	522.81±15.73a
Root	MS-N1	1.43±0.16a	1.96±0.08a	3.53±0.07a	7.63±0.46a	20.15±1.04b
	SB1-N1	1.30±0.14a	1.95±0.16a	4.28±0.19a	9.16±0.34a	25.92±1.31a
	SB2-N1	1.23±0.08a*	1.94±0.07a	4.27±0.33a	8.01±0.14a	24.03±0.64ab
	MS-N2	1.14±0.07b	2.77±0.21ab	4.17±0.24a	8.26±0.31a	21.33±0.43a
	SB1-N2	1.55±0.14a	3.11±0.19a	4.22±0.13a	9.52±0.69a	26.48±0.95a
	SB2-N2	1.81±0.12a	2.20±0.15b	4.41±0.26a	8.55±0.80a	24.97±1.06a

Note: Different uppercase letters indicate significant (p<0.05) differences between different cropping patterns at the N1 level, different lowercase letters indicate significant (p<0.05) differences be-tween different cropping patterns at the N2 level, and * indicates significant (p<0.05) differences between N1 and N2 in the same cropping pattern.

Table S2. Effects of planting pattern and N application rate on the shoot and root C amount (g·m^{-2}) of soybean at different stages in 2021

		seedling stage	flowering stage	maturity stage
Shoot	SB1-N1	1.03±0.08a	5.7±0.56a	23.58±1.02a
	SB2-N1	0.79±0.05b	5.74±0.51a	22.68±1.4a
	SB1-N2	0.81±0.03a	6.39±0.43a	23.26±1.1a
	SB2-N2	0.68±0.08a	6.48±0.7a	23.51±2.93a
Root	SB1-N1	0.1±0.01a	0.42±0.03a	4.37±0.39a
	SB2-N1	0.09±0.01a	0.39±0.01a	3.63±0.89a
	SB1-N2	0.09±0.01a	0.46±0.02a	3.44±0.16a
	SB2-N2	0.1±0.01a	0.4±0.04a	3.49±0.23a

Note: Different uppercase letters indicate significant (p<0.05) differences between different cropping patterns at the N1 level, different lowercase letters indicate significant (p<0.05) differences be-tween different cropping patterns at the N2 level, and * indicates significant (p<0.05) differences between N1 and N2 in the same cropping pattern.

Table S3. Effects of planting pattern and N application rate on the shoot and root C amount (g·m^{-2}) of system at different sugarcane growth stages in 2021

		sugarcane germination stage	sugarcane seeding stage	sugarcane tillering stage	sugarcane jointing stage	Sugarcane maturity stage
Shoot	MS-N1	10.9±1.12c	38.44±1.44b	141.69±5.69b	1080.26±80.03b	2803.24±76.2b
	SB1-N1	18.01±0.71a	82.14±4.67a	307.07±9.54a	1315.76±41.21a	3027.69±45.64a
	SB2-N1	15.04±0.61b	88.58±4.19a	305.45±17.35a	1337.07±32.72a	3050.55±56.89a
	MS-N2	11.13±1.00b	45.95±1.04b	152.66±6.02b	1193.59±102.88a	2907.45±47.92a
	SB1-N2	16.27±0.76a	84.23±0.36a	300.32±9.20a	1343.45±111.24a	3075.87±47.15a
	SB2-N2	16.49±1.42a	81.69±2.33a	296.93±17.77a	1339.54±2.32a	3010.1±90.55a
Root	MS-N1	8.23±0.92b	11.26±0.43b	20.32±0.42b	43.92±2.67a	116.02±5.97b
	SB1-N1	8.06±0.76a	13.64±0.81a*	49.46±3.06a	52.74±1.94a	149.22±7.56a
	SB2-N1	7.61±0.4a*	13.41±0.43a	45.2±6.35a	46.09±0.79a	138.33±3.67a
	MS-N2	6.58±0.43ab	15.96±1.23b	24.01±1.38b	47.55±1.81a	122.78±2.46b
	SB1-N2	9.42±0.82a	20.54±1.13a	43.86±1.30a	54.81±3.95a	152.47±5.45a
	SB2-N2	10.95±0.73a	14.96±1.04b	45.21±1.86a	49.25±4.60a	143.75±6.13a

Note: Different uppercase letters indicate significant (p<0.05) differences between different cropping patterns at the N1 level, different lowercase letters indicate significant (p<0.05) differences be-tween different cropping patterns at the N2 level, and * indicates significant (p<0.05) differences between N1 and N2 in the same cropping pattern.

Table S4. Effects of planting pattern and N application rate on the soil TOC content ($g\cdot kg^{-1}$) at different sugarcane growth stages in 2021

	sugarcane germination stage	sugarcane seeding stage	sugarcane tillering stage	sugarcane Jointing stage	sugarcane maturity stage
MS-N1	9.63±0.74a	10.62±0.87a	11.44±0.83b	11.45±0.53b	8.39±0.70b
SB1-N1	10.14±0.70a	11.06±0.58a	12.79±0.86ab	13.75±1.02a	10.79±1.04a
SB2-N1	10.25±0.40a	11.99±0.94a	13.04±0.33a	14.14±0.34a	9.57±0.95ab
MS-N2	10.38±0.42a	10.94±0.91b	11.86±0.60a	12.52±1.42a	8.69±0.89a
SB1-N2	11.61±0.63a	12.75±0.81a	12.55±0.64a	14.48±0.80a	9.91±0.46a
SB2-N2	10.58±0.91a	11.27±1.33ab	11.69±0.70a	13.94±0.93a	9.60±0.46a

Note: Different uppercase letters indicate significant (p<0.05) differences between different cropping patterns at the N1 level, different lowercase letters indicate significant (p<0.05) differences be-tween different cropping patterns at the N2 level, and * indicates significant (p<0.05) differences between N1 and N2 in the same cropping pattern.

Table S5. Effects of planting pattern and N application rate on the soil MBC content ($mg \cdot kg^{-1}$) at different sugarcane growth stages in 2021

	sugarcane germination stage	sugarcane seeding stage	sugarcane tillering stage	sugarcane Jointing stage	sugarcane maturity stage
MS-N1	37.95±3.24b	60.41±3.56b	99.53±5.49b	92.59±10.27b	75.6±7.52a
SB1-N1	49.99±1.37a	78.59±8.95a	120.49±6.45a	114.58±8.46a	85.00±4.82a
SB2-N1	51.23±1.35a	77.23±4.71a	119.24±6.09a	111.16±7.82a	82.67±7.96a
MS-N2	41.7±7.21a	68.81±7.38a	108.15±11.27a	104.03±7.08a	65.33±5.03b
SB1-N2	44.26±4.61a	73.66±7.14a	112.32±5.67a	118.28±12.39a	74.69±4.29a
SB2-N2	44.55±5.4a	74.79±5.07a	114.66±13.3a	110.44±7.59a	71.43±5.70ab

Note: Different uppercase letters indicate significant ($p<0.05$) differences between different cropping patterns at the N1 level, different lowercase letters indicate significant ($p<0.05$) differences be-tween different cropping patterns at the N2 level, and * indicates significant ($p<0.05$) differences between N1 and N2 in the same cropping pattern.

Table S6. Effects of planting pattern and N application rate on the soil DOC content ($mg \cdot kg^{-1}$) at different sugarcane growth stages in 2021

	sugarcane germination stage	sugarcane seeding stage	sugarcane tillering stage	sugarcane jointing stage	sugarcane maturity stage
MS-N1	35.81±1.19b	37.01±1.93b	51.73±6.65a	53.37±3.28b	51.73±6.65a
SB1-N1	36.78±1.50b	50.67±4.44a	54.90±3.16a	67.6±2.81a	54.90±3.16a
SB2-N1	43.19±2.16a	52.74±3.29a	54.97±3.06a	63.87±6.42a	54.97±3.06a
MS-N2	32.86±1.89a	40.90±1.59b	50.45±2.68a	50.82±2.00b	50.45±2.68a
SB1-N2	37.05±2.13a	45.19±3.57ab	52.02±5.61a	60.13±2.03a	52.02±5.61a
SB2-N2	35.23±2.20a	50.39±2.52a	53.54±2.81a	53.33±5.50b	53.54±2.81a

Note: Different uppercase letters indicate significant (p<0.05) differences between different cropping patterns at the N1 level, different lowercase letters indicate significant (p<0.05) differences be-tween different cropping patterns at the N2 level, and * indicates significant (p<0.05) differences between N1 and N2 in the same cropping pattern.

Table S7. Effects of planting pattern and N application rate on the soil nutrient content in 2021

	Total N ($g·kg^{-1}$)	Total P ($g·kg^{-1}$)	Total K ($g·kg^{-1}$)	Alkaline hydrolysis N ($mg·kg^{-1}$)	Ammonium N ($mg·kg^{-1}$)	Nitrate N ($mg·kg^{-1}$)
MS-N1	0.58±0.03a	0.79±0.07a	21.61±0.77a	75.29±5.11b	9.73±0.30b	22.1±1.49b*
SB1-N1	0.60±0.02a	0.78±0.04a	21.29±0.83a	112.12±9.81a	13.73±0.37a	47.58±1.57a
SB2-N1	0.69±0.03a	0.8±0.03a	20.89±1.07a	101.35±13.41ab*	14.38±0.75a	45.16±4.44a
MS-N2	0.54±0.03a	0.78±0.02a	21.35±0.68a	81.39±7.29b	11.05±0.60b	38.85±1.46b
SB1-N2	0.61±0.02a	0.71±0.11a	22.24±0.71a	132.16±9.30a	14.38±0.33a	50.31±1.20a
SB2-N2	0.61±0.03a	0.83±0.05a	22.00±0.57a	139.54±2.51a	13.78±0.88a	54.97±2.76a

Note: Different uppercase letters indicate significant (p<0.05) differences between different cropping patterns at the N1 level, different lowercase letters indicate significant (p<0.05) differences be-tween different cropping patterns at the N2 level, and * indicates significant (p<0.05) differences between N1 and N2 in the same cropping pattern.

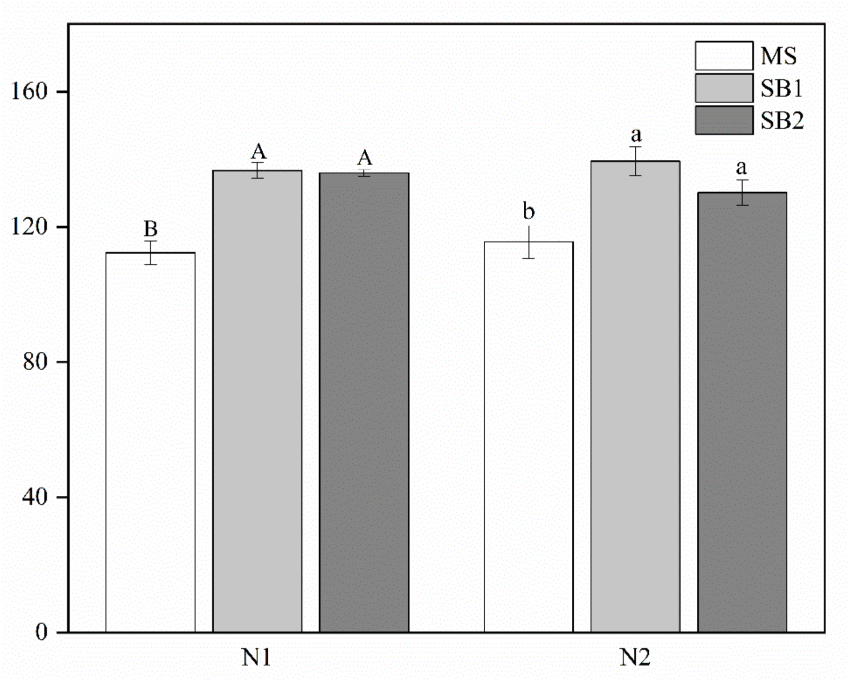

Figure S1. Effects of planting pattern and N application rate on sugarcane yield in 2021. Different uppercase letters indicate significant (p<0.05) differences between different cropping patterns at the N1 level, different lowercase letters indicate significant (p<0.05) differences between different cropping patterns at the N2 level, and * indicates significant (p<0.05) differences between N1 and N2 in the same cropping pattern.
